# Use of Arsenic in Phthisis

**Published:** 1871-01

**Authors:** 


### THERAPEUTICAL USE OF ARSENIC IN PHTHISIS.

Under the care of Dr. Nonat, at the Hospital la Charite, Paris.

On a recent visit to the Charite, Dr. Nonat kindly communicated to us his experience of the therapeutical effects of arsenic in Phthisis. The very favorable results which Dr. Moutard-Martin of Beaujon Hospital, had derived from arsenic in the treatment of tuberculosis, had led M. Nonat to try the substance in a large number of cases. He has administered the remedy under the form of arsenious acid, and in doses of one milligramme, in pills to begin with. The dose was gradually increased every eight days by one milligramme, till he would reach the dose of four milligrammes a day. In these conditions the medicament has afforded him good results in cases where tuberculosis had attained only the first or second stage, and presented no intestinal complication, for when vomiting and diarrhœa have set in, arsenic must be at once discarded. When phthisis is incipient, and when it is well circumscribed, M. Nonat has seen arsenic increase the appetite and strength of the patients. They gain flesh, look much better, and feel stronger and more cheerful. In such cases the medicament does not increase the pulmonary congestion, and indeed is attended by no inconvenience. The only counter-irritation lies in the alimentary canal. In many subjects, however, placed in the above conditions, arsenic, if it did no harm, failed to produce any benefit.

Dr. Nonat's rule for the employment of arsenic is, therefore, to administer it only in cases of well-circumscribed phthisis. When tuberculization is generalized, or far-advanced, arsenic, far from producing any good, increases the irritation of the bowels, and brings on gastro-intestinal disorder: the local action of the substance becomes noxious.

Summing up the practical results of his experience of arsenic, M. Nonat says that good effects, in hospital, have been the exception in patients in whom the disease has attained an advanced stage; but in civil or town practice, where the physician is consulted at an earlier period, the results have been good in a large number of cases. In a word, M. Nonat believes that arsenic is a remedy which must not be neglected in the incipient stage of phthisis, or when the disease is well circumscribed; but he adds that its virtue has been too highly extolled.—*Half-Yearly Abstract.*

